# Synthesis and spectroscopic properties of 4-amino-1,8-naphthalimide derivatives involving the carboxylic group: a new molecular probe for ZnO nanoparticles with unusual fluorescence features

**DOI:** 10.3762/bjoc.9.147

**Published:** 2013-07-03

**Authors:** Laura Bekere, David Gachet, Vladimir Lokshin, Wladimir Marine, Vladimir Khodorkovsky

**Affiliations:** 1 Aix Marseille Université, CNRS, CINaM UMR 7325, 13288, Marseille, France

**Keywords:** fluorescence, hydrogen bond, 1,8-naphthalimide, one- and two-photon absorption, zinc oxide

## Abstract

Of a series of 4-substituted 1,8-naphthalimides, fluorescent 4-(6-piperidinyl-1,3-dioxo-1*H*-benzo[*de*]isoquinolin-2(3*H*)-yl)benzoic acid (**4**) was found to be a sensitive molecular probe for ZnO nanoparticles. We investigated in detail one- and two-photon absorption properties of this fluorophore. In nonpolar solvents, the acid **4** absorbs at about 400 nm and fluoresces at 500 nm with a fluorescence lifetime of about 7 ns, similar to the ester **6** and typical of the lifetimes of other derivatives of this type. Although the anionic form of this acid is not fluorescent, partial ionization of **4** in polar solvents, such as ethanol and acetonitrile, is not only accompanied by the expected decrease in the fluorescence quantum yield, but also gives rise to bathochromic shifts of both absorption and fluorescence and dual fluorescence with lifetimes of 0.2–0.3 ns and 6 ns ascribed to the formation of anionic complexes. The interaction with the ZnO surface brings about further considerable changes in the fluorescence patterns.

## Introduction

1,8-Naphthalimide derivatives are useful for a variety of applications owing to their strong fluorescence, electroactivity and photostability. These compounds can be employed as dyes for natural and synthetic fibers [1], optical brighteners in detergents, textiles, polymeric materials [1–3] and chemiluminiscent agents [4]. Owing to the high fluorescence quantum yields 1,8-naphthalimides are used as laser dyes [5–6]. Some naphthalimide derivatives have also been reported to be efficient fluorescent molecular probes [7–8].

Recently, the potential of amino-1,8-naphthalimides as fluorescent sensors in living cells has been reviewed [9]. Dendrimers involving the amino-1,8-naphthalimide moieties were showed to be promising active nondoping emitters [10]. 4-Amino-substituted naphthalimide dyes are important yellow components of daylight fluorescent pigments and can be employed as fluorescent dichroic dyes in liquid-crystal displays [1–2,11].

In this paper, we report on the synthesis [12], characterization and one- and two-photon spectroscopic properties of previously unknown methyl 4-(6-piperidinyl-1,3-dioxo-1*H*-benzo[*de*]isoquinolin-2(3*H*)-yl)benzoate and the corresponding acid. The phenyl moiety can serve as a rigid spacer, while the carboxylic group can function as an anchor for grafting the fluorophore to metal oxide surfaces. This approach to the modification of ZnO nanoparticles produced by femtosecond laser ablation was demonstrated to afford nanohybrid materials for biophotonic applications [13–14].

## Results and Discussion

### Synthesis and characterization

Yellow 4-amino-1,8-naphthalimides are usually synthesized in two steps. The first step is the condensation of 4-chloro-1,8-naphthalic anhydride with amines in 1,4-dioxane or 2-methoxyethanol under reflux yielding the corresponding 4-chloro-1,8-naphthalimides. The second step involves the substitution of the chlorine atom with aliphatic primary or secondary amines.

**Scheme 1:**
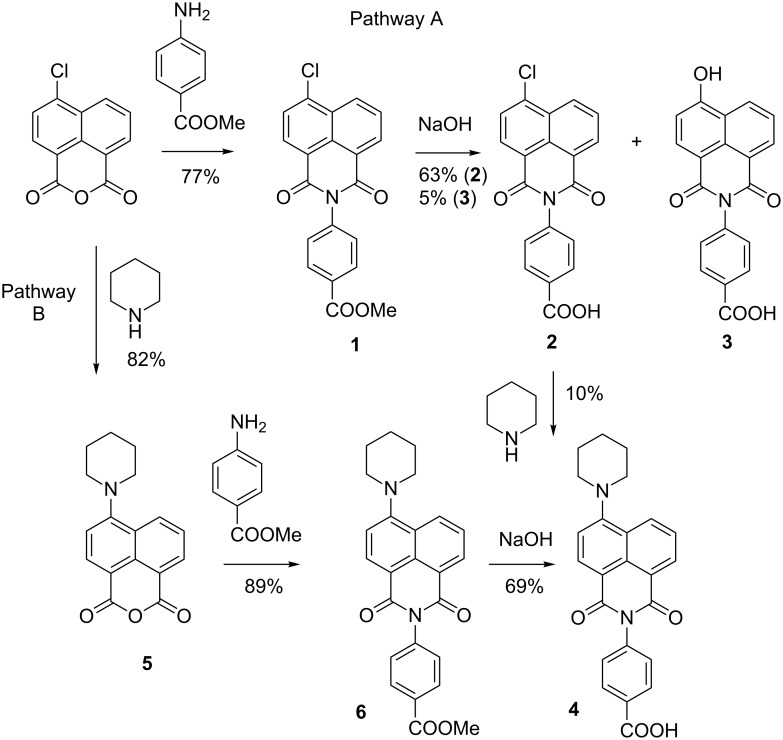
Synthesis and structures of 1,8-naphthalimides.

Our target derivative was designed to involve the benzoic acid moiety at the N-atom of the 1,8-naphthalimide. At first we used a general straightforward method involving the hydrolysis of the chloro substituted imide **1** and eventual substitution of the chlorine atom by piperidine. (Scheme 1, Pathway A). The pathway A has two major inconveniences. Hydrolysis of intermediate **1** was successful only under the basic conditions, since even prolonged heating of **1** under reflux with a strong mineral acid (HCl or H_2_SO_4_) afforded no expected product **2**. A mixture of 6-chloro- and 6-hydroxy-derivatives **2** and **3** were obtained upon treatment with a small excess of sodium hydroxide. Pure 6-hydroxy derivative **3** can be obtained in the reaction of ester **1** with 4 equivalents of NaOH in boiling water. The reaction of acid **2** with piperidine proceeded slowly and was accompanied by the formation of a number of unidentified byproducts.

To avoid side reactions, we tried another approach that appeared to be effective. The chlorine atom was replaced with the piperidine moiety in the first step (Scheme 1, Pathway B). 4-Piperidinyl-1,8-naphthalic anhydride **5** [1] was condensed with methyl 4-aminobenzoate and the corresponding ester **6** was isolated in high yield. The target acid **4** was obtained in 69% yield by hydrolysis of **6** under basic conditions. The reactions were monitored by thin-layer chromatography (TLC) on silica gel eluted with CH_2_Cl_2_ or EtOH/CH_2_Cl_2_. All synthesized new compounds were characterized by the ^1^H NMR spectra and the elemental analyses.

### Photophysical properties

The spectroscopic properties of 1,8-naphthalimides are strongly dependent on the substituent at C-4 of the naphthalene ring. In general, the derivatives with a halogen atom or alkoxy groups are colorless and exhibit blue fluorecence [15–16], while the amino-substituted 1,8-naphthalimides are yellow and exhibit green fluorescence [17].

**Figure 1:**
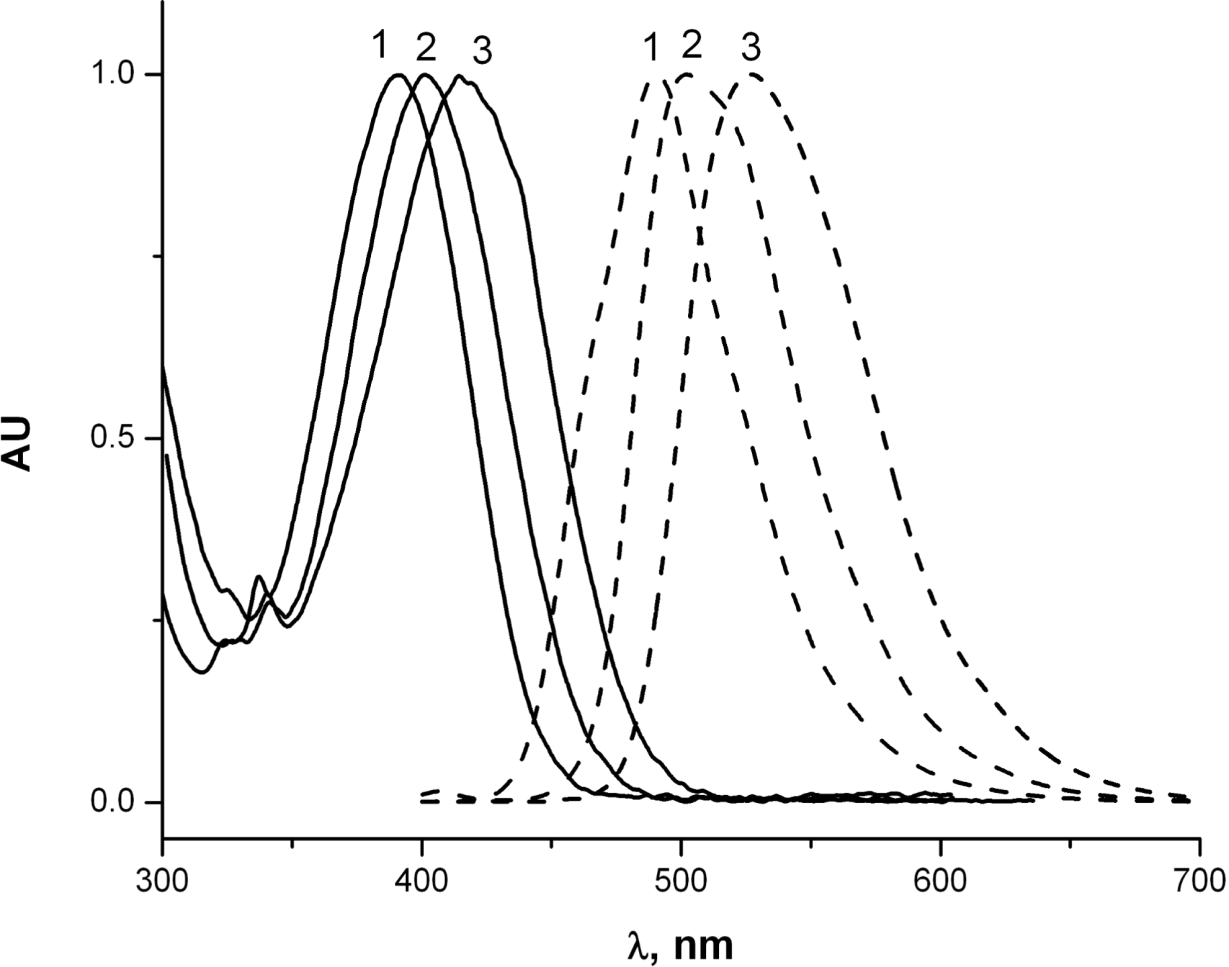
Normalized absorption (solid curves) and fluorescence (dashed curves) spectra of **4**: (1) in cyclohexane; (2) in toluene; and (3) in CH_2_Cl_2_.

**Figure 2:**
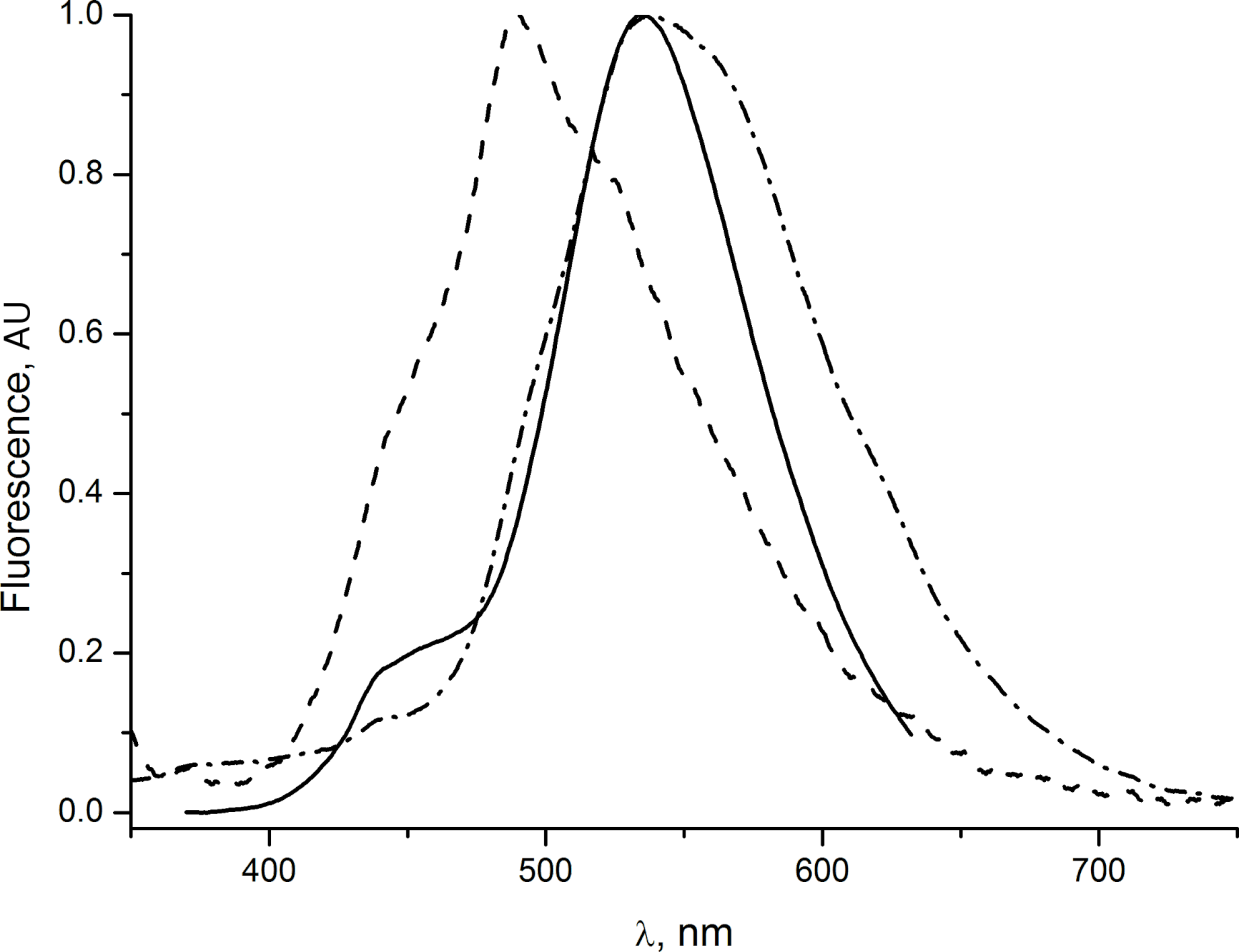
Normalized fluorescence spectra of **4** in ethanol (5 × 10^−5^ M): (solid line) neutral solution; (dashed) acidified solution; (dashed-dot) basic solution.

**Figure 3:**
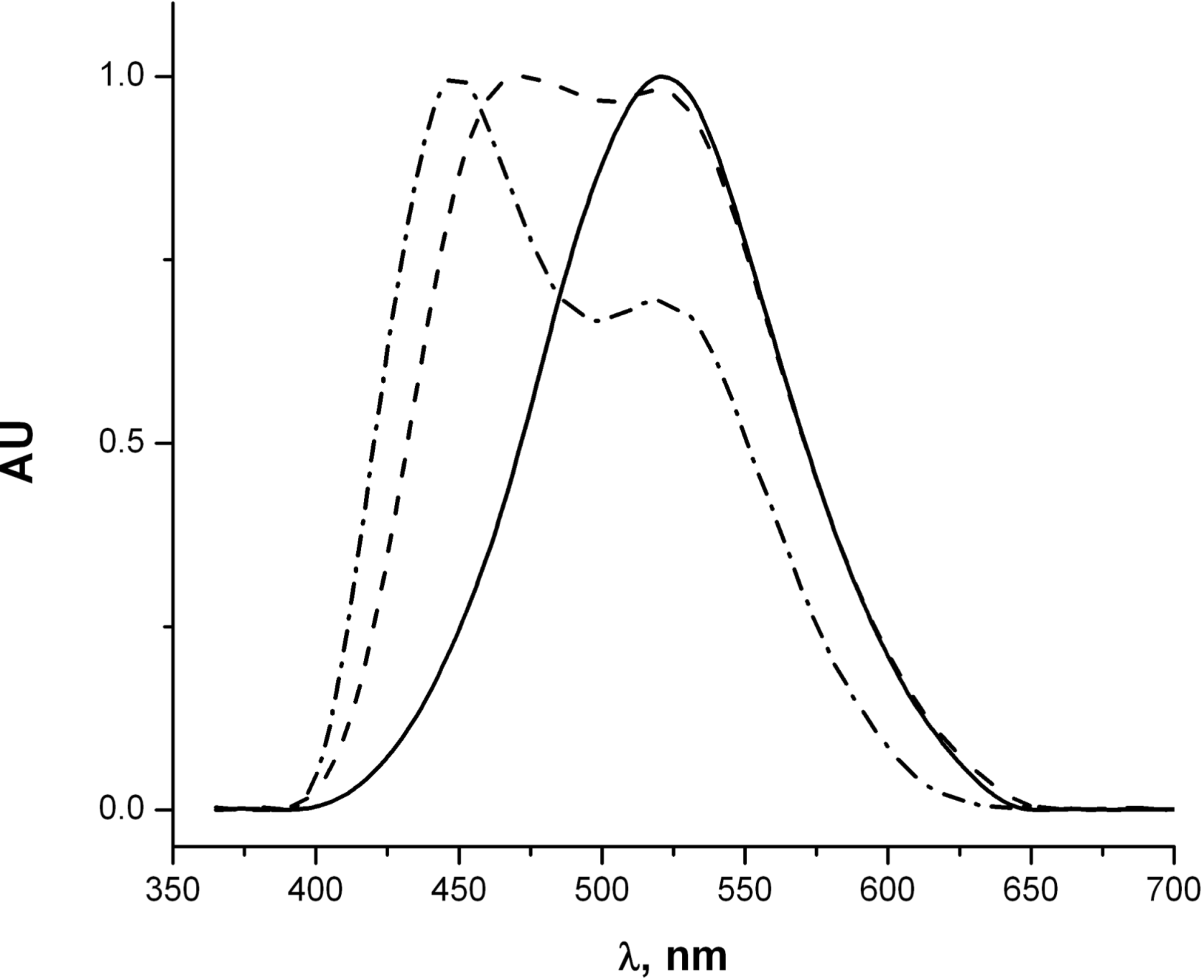
Normalized fluorescence spectra of **4** in ethanol at different concentrations: (solid line) 10^−4^ M; (dashed line) 10^−5^ M; (dashed-dot) 10^−6^ M.

Derivatives **1, 2, 3, 4** and **6** were characterized by one-photon absorption and fluorescence spectroscopy (Table 1). In nonpolar, as well as in polar solvents, chloro-substituted intermediates **1** and **2** exhibit absorption maxima at 341 and 345 nm and fluorescence maxima at 397 and 401 nm, respectively. In contrast, the spectroscopic properties of derivatives **3** and **4** depend on the solvent polarity. The absorption and fluorescence maxima of **4** in different solvents are collated in Table 2 and the typical spectra are shown in Figure 1. The observed bathochromic shifts are moderate and the strongest shifts in absorption (in ethanol) and fluorescence (in acetonitrile) can be related to partial ionization of the acids **3** and **4** in these solvents. The pure sodium salt of **4** is not fluorescent and absorbs at 322 nm (in water). At the same time, the addition of a few drops of aqueous HCl shifts the fluorescence maximum to 490 nm and the addition of a few drops of ethanolic NaOH brings about a red shift and broadening of the fluorescence band (Figure 2). Moreover, upon dilution of a solution of **4** in ethanol, the shoulder observed at about 450 nm at the concentration of 5 × 10^−5^ M increases and becomes the major band at lower concentrations as shown in Figure 3.

**Table 1:** Optical properties of derivatives **1**–**4**, **6** in ethanol.

Compound	λ_abs_ max	λ_flu_ max	ε, l mol^−1^ cm^−1^

**1**	341	397	17 700
**2**	345	401	15 000
**3**	342	415	16 000
**4**	417	535	13 400
**6**	415	533	12 100

**Table 2:** Solvent-dependent optical properties of the dye **4**.

Solvent	λ_abs_ max	λ_fl_ max

Cyclohexane	391	490
Toluene	401	500
CHCl_3_	415	519
THF	405	523
CH_2_Cl_2_	414	528
EtOH	417	535
Acetonitrile	412	540

**Scheme 2:**
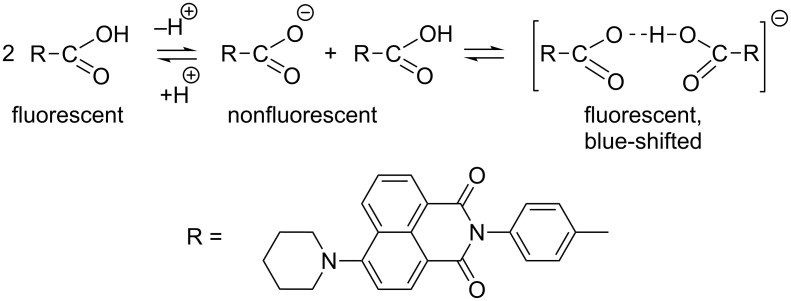
Possible ionization and complexation of **4** in ethanol.

The acid **4** and its ester **6** show strong fluorescence in nonpolar solvents such as cyclohexane and toluene (quantum yield for **4** is 75 ± 10%), while in polar solvents such as ethanol, the fluorescence intensity of **4** considerably diminishes (quantum yield ≈10%). This phenomenon can also be related to partial ionization: the formation of the nonfluorescent anionic species and fluorescent anionic complex in ethanol, as shown in a simplified form (the potential existence of the acid dimers is omitted) in Scheme 2. We exclude the possibility of participation of the amino group in the equilibrium since, according to our preliminary experiments, the parent compound prepared from 1,8-naphthalic anhydride behaves similarly.

**Figure 4:**
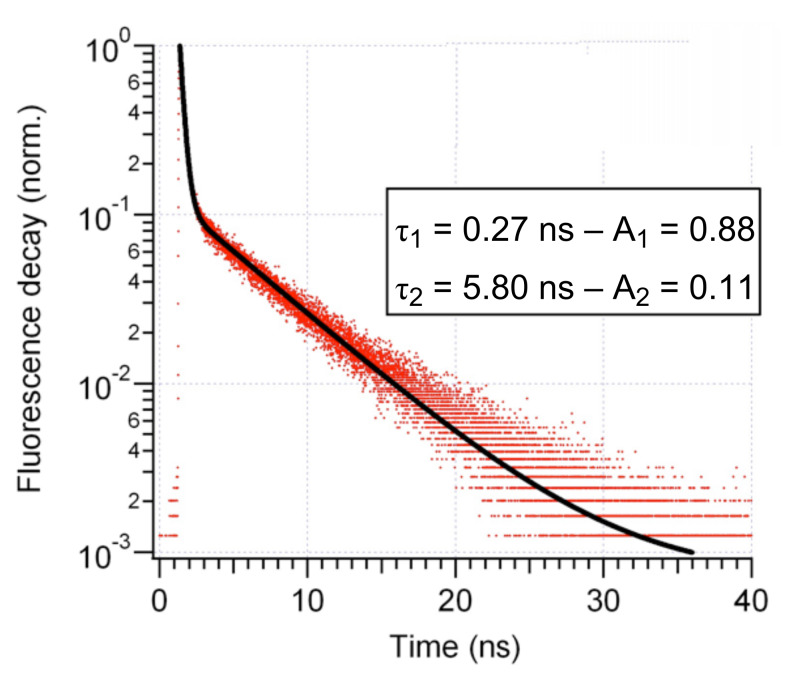
Fluorescence decay profile of **4** from a 10^−5^ M solution in ethanol (excitation at 450 nm, detection at 550 nm).

The fluorescence lifetime measurements were performed under 350–450 excitation and detection between 470 and 700 nm in toluene and ethanol. We observed a strong influence of the solvent on the photophysical properties of compound **4**. In toluene, at 500 nm (corresponding to the fluorescence maximum), the fluorescence decay followed a mono-exponential law with a lifetime equal to 6.98 ns. This value is comparable with the lifetimes of other 4-amino substituted naphthalimides without the carboxylic group in ethanol (7.5–10.5 ns [17]). In ethanol, measurements of fluorescence decay time show the complex nature of the deactivation of the excited states. Only excitation at short wavelength, 350–360 nm, and detection at the blue edge of the emission spectra, 455–470 nm, shows a single exponential decay with lifetime of about 5.77 ± 0.2 ns. The shifts of the excitation wavelength to the red inevitably induce a bi-exponential decay with very strong nonradiative component. A typical decay curve is shown in Figure 4. Fitting of the experimental data gives fast decay, 0.21–0.3 ns (amplitude 0.95–0.85), depending on the detection wavelength with a subsequent slow component with small amplitude of about 0.1 and a typical lifetime between 5.8–6.02 ns (detection at 550 nm) depending on the dye concentration. A Stern–Volmer plot shows a simultaneous static and dynamic quenching, probably superimposed with FRET between pure acid **4** and the anionic complex, formed in ethanol. The full analysis of the formation of these complexes will be published elsewhere.

**Figure 5:**
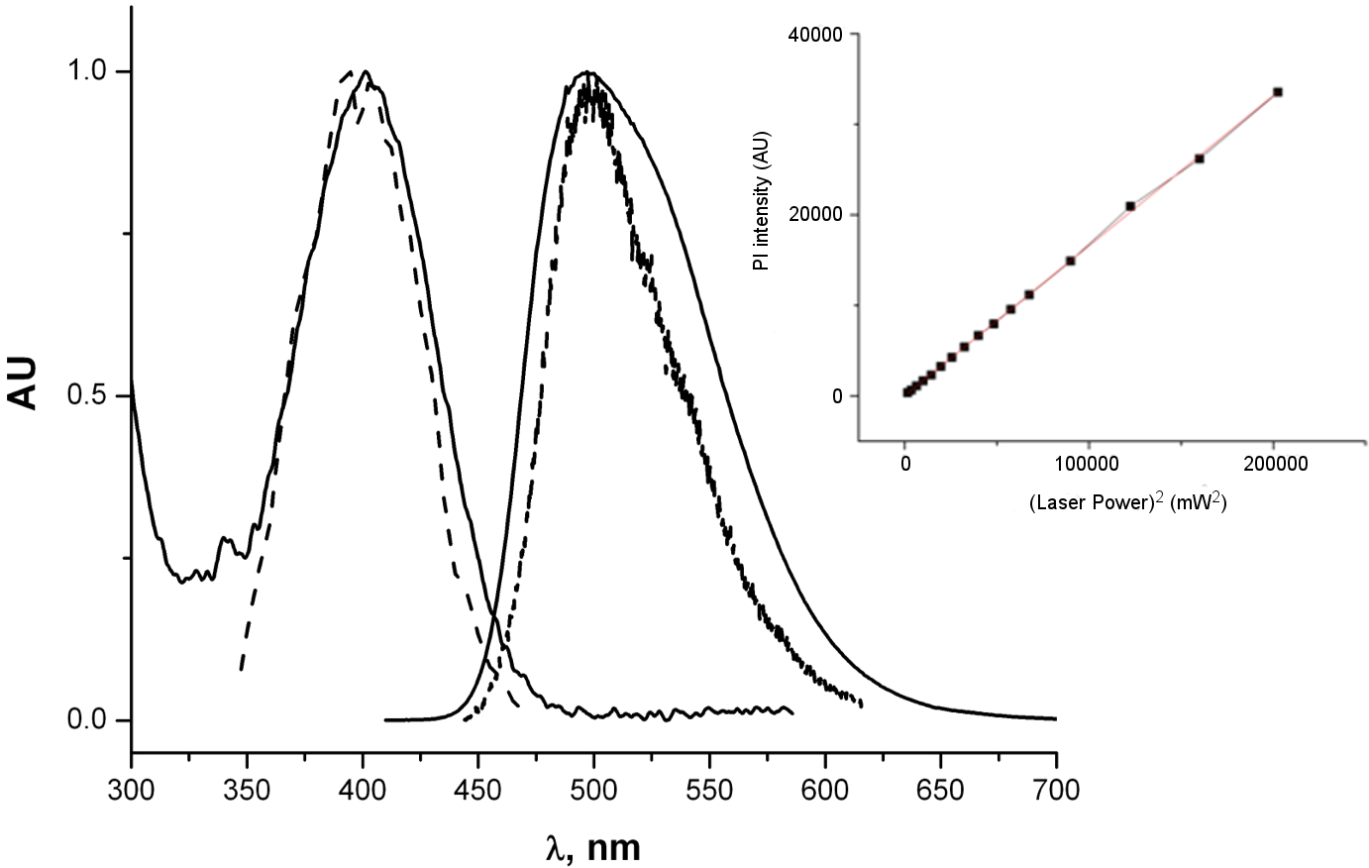
Normalized one- and two-photon spectra of dye **4** in toluene. Solid curves: one-photon absorption and fluorescence; dashed curves two-photon fluorescence excitation (the X-axis values are λ/2) and fluorescence spectra. Inset: fluorescence intensity versus (laser power)^2^ at 800 nm.

The two-photon absorption cross section of **4** in toluene was found to amount to about 80 GM at 800 nm. The two-photon fluorescence excitation and fluorescence spectra are somewhat narrower than the respective one-photon spectra (Figure 5), but seem to correspond to the same electronic transitions. The dependence of the fluorescence intensity on the square of the laser power shown in the inset (Figure 5) clearly demonstrates the two-photon excitation nature.

### Quantum mechanical calculations

Quantum mechanical calculations were performed for derivative **4** to gain deeper insight into the nature of the longest wavelength transition and to estimate the electron donating and accepting properties of this molecule. The geometry was optimized by using the semiempirical AM1 method and the electronic spectra were calculated by using the TD B3LYP/6-31G(d,p) method. The ionization potential (IP) and electron affinity (EA) were calculated at the B3LYP/6-31G(d,p) level by using the energies of the corresponding radical cation and anion.

**Figure 6:**
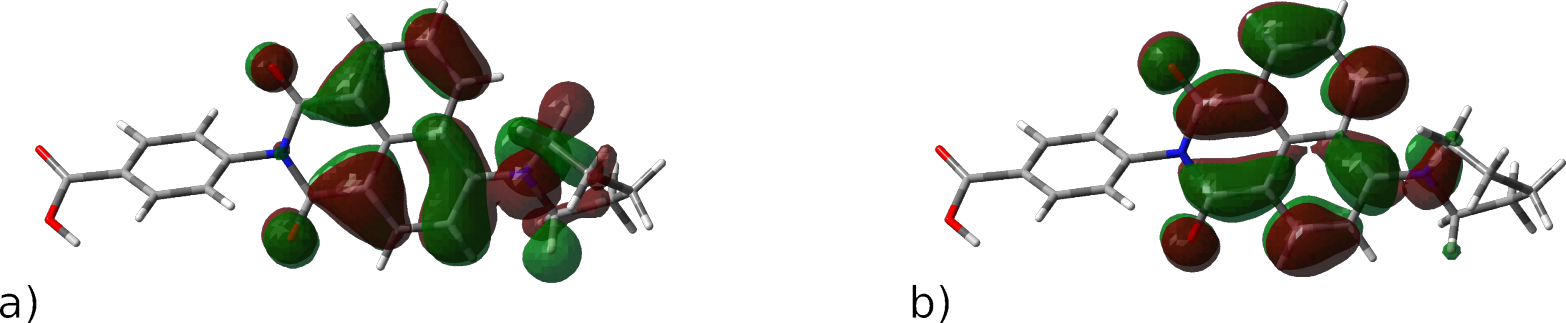
(a) HOMO and (b) LUMO of **4**.

Thus, the first electronic transition is predicted at 401 nm (f = 0.30). It is essentially the HOMO → LUMO transition. The next five electronic transitions between 333 and 300 nm are weak (f = 0.001–0.003) and involve mostly the orbitals localized within the naphthalene and phenyl rings. These results are in a good agreement with the experiment (Figure 1). It is noteworthy that both HOMO and LUMO orbitals are delocalized and involve both the naphthalene moiety and the amino substituent (Figure 6a,b). In addition, the phenyl ring is twisted out of the molecule plane and does not contribute to either orbital. The degree of charge transfer is not large, which accounts for the relatively moderate solvatochromism. The calculated IP and EA values are 7.13 and 0.78 eV, respectively. Derivative **4** should thus exhibit stronger electron-donating properties and weaker electron-accepting properties than ZnO [18].

### Grafting to the surface of ZnO nanoparticles

**Figure 7:**
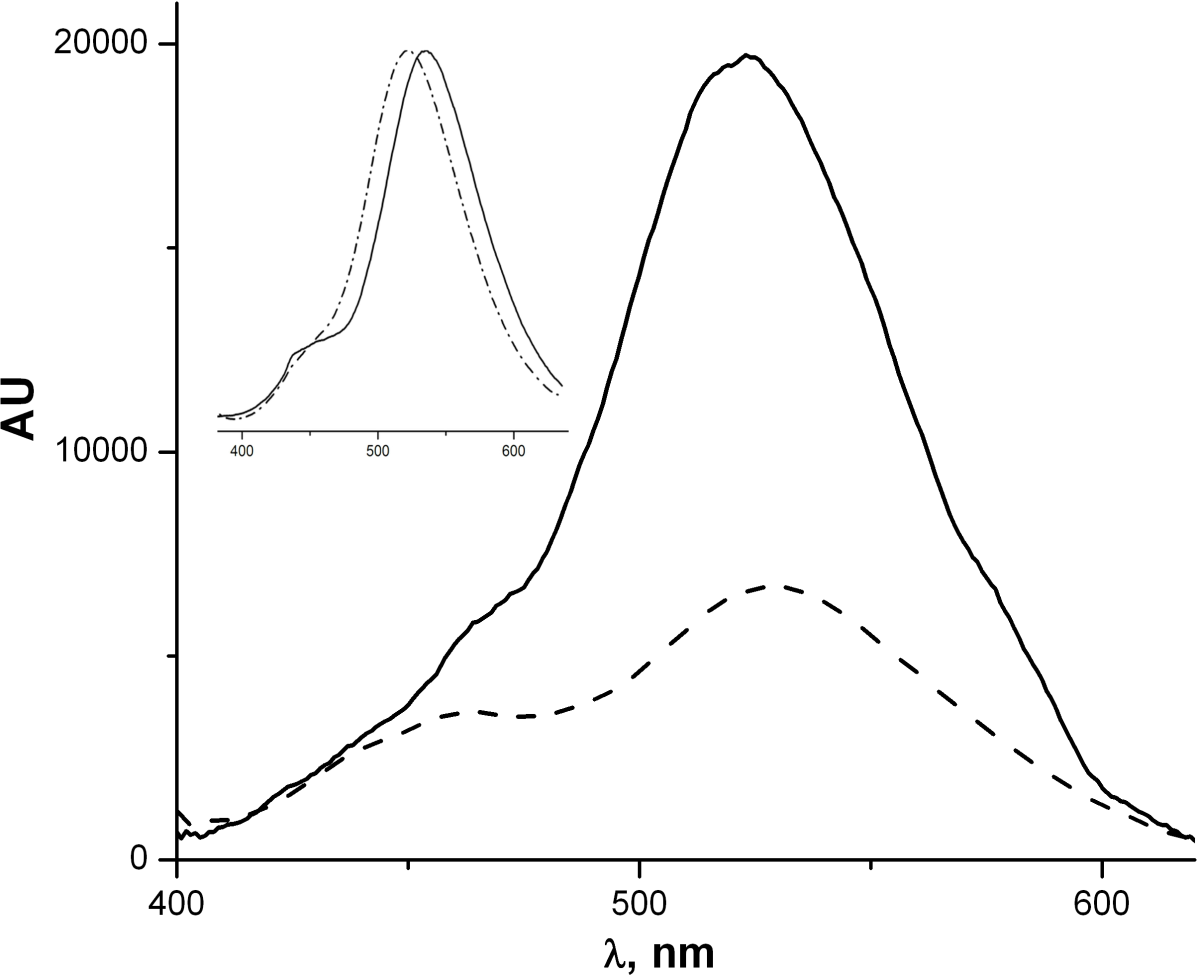
Fluorescence spectra of **4** ethanol (5 × 10^−5^ M) (dashed curve) before and after the addition of ZnO NPs (solid curve). Excitation wavelength: 325 nm. The inset shows the same normalized spectra.

In order to investigate the behavior of acid **4** as a fluorescent molecular probe, preliminary experiments on grafting to the surface of ZnO nanoparticles (NPs) were carried out. The ZnO NPs in ethanol were prepared by laser ablation according to [14]. The typical NP size was 17 nm with the dispersion about 25%. A freshly prepared ZnO NP solution in ethanol was added to the 1.4 × 10^−6^ M solution of **4** in ethanol. Under one-photon CW excitation at 325 nm, the fluorescence intensity of the solution exhibited a four-fold enhancement. A 14 nm blue-shift was also observed (Figure 7). Fluorescence from **4** persists after several centrifugation/washing cycles (which completely remove **4** from the solvent) and the band shape does not undergo further change, thus proving the ZnO surface grafting.

## Conclusion

Derivative **4** involving both the fluorophore moiety and the carboxylic group capable of binding the ZnO surface can serve as an efficient molecular probe owing to the high sensitivity to the polarity of the surroundings. Derivative **2** can also be used when fluorescence detection at shorter wavelengths is desired. Moreover, owing to the absence of fluorescence from the anionic species these compounds can be employed for monitoring the complex equilibria of carboxylic acids existing in polar solvent solutions. The detailed quantitative investigation of the behavior of derivatives **2** and **4** and the unsubstituted parent derivative is underway in our laboratory.

## Experimental

All starting materials: 4-chloro-1,8-naphthalic anhydride, piperidine, methyl 4-aminobenzoate and solvents were obtained from Alfa Aesar. 4-Chloro-1,8-naphthalic anhydride and methyl 4-aminobenzoate were crystallized from acetic anhydride and methanol, respectively. Distilled water was used in all experiments.

^1^H NMR spectra were recorded on a Brucker AC 250 spectrometer. Proton chemical shifts are reported in ppm downfield from tetramethylsilane. The elemental analyses were made by the Microanalytical Center of Aix-Marseille Université. Melting points were determined on a Büchi-510 apparatus and were not corrected. The UV–vis absorption spectra were recorded with an Ocean Optics USB 4000 spectrometer. The fluorescence spectra were recorded with an HORIBA Fluorolog FL-1057 spectrometer. All spectra were recorded using 1 cm quartz cells.

The quantum yield of fluorescence was determined by using coumarin 153 as a reference in toluene and ethanol at 20 °C.

Fluorescence lifetime measurements were performed by using time-correlated single photon counting (TCSPC) spectroscopy. Femtosecond pulses (150 fs, 80 MHz) generated by a tunable mode-locked Ti:sapphire laser (MaiTai HP, Spectra-Physics, 690–1050 nm) were externally frequency-doubled by a 1 mm thick BBO crystal. The pulse repetition rate was reduced to 8 MHz by the means of a pulse-picker (Model 3980, Spectra-Physics), extending the delay between the consecutive pulses to 126 ns. The generated femtosecond pulses illuminated a 1 cm quartz cell containing the solution under study. The photo-excited fluorescence was collected in a 90° geometry and sent into a subtractive double-monochromator (DH10, Jobin-Yvon) before being detected by a silicon avalanche photodiode (PDM50CTC, Micro Photon Devices). The entrance and exit slit widths of the monochromator ensured 1 nm spectral resolution in the lifetime measurements. Depending on the experiment, a small part of the 700 or 800 nm laser beam was picked and sent to a photodiode (TDA 200, Picoquant). The laser and fluorescence signals were subsequently sent to a correlator (PicoHarp 300, Picoquant). The time of arrival of the fluorescence photons was measured with 4 ps or 16 ps resolution depending on the experiment, and the fluorescence temporal decay was reconstructed. The impulsive response function (IRF) was taken as the decay curve at the excitation wavelength (400 nm). The IRF curve was free from the fluorescence signal. From the IRF curves, we estimated the temporal response of the setup as 150 ps FWHM. The data were numerically deconvoluted using the measured IRF and fitted to mono- or bi-exponential decays, using a commercial program (IgorPro, Wavemetrics).

The two-photon absorption cross section was determined by using fluorescence excitation spectroscopy. The same tunable femtosecond laser source (operating at 80 MHz), was used to excite a solution of **4** by two-photon absorption. The laser beam was spatially extended with a telescope and was focused into the 1 cm cell containing a solution of **4** in toluene with a NA 0.4 objective lens. The generated fluorescence was collected at 90° and filtered through a short-pass interference filter. After passing through a monochromator (Spectra Pro 500i, Acton) it was detected by a CCD camera (Roper Scientific). The fluorescence spectra were recorded for the excitation wavelengths between 700 to 950 nm, in 5 nm steps. Integration time ranged from 10 ms to 2 s depending on the fluorescence signal level. The average laser power was typically kept around 500 mW. The two-photon absorption spectra were calculated by integrating the fluorescence spectra for each excitation wavelength. The spectra were normalized by the integration time and the square of the average laser power. The two-photon absorption cross section of **4** was determined by comparing the two-photon absorption spectrum with that of rhodamine B (RhB) in methanol, and by normalizing by its quantum yield. The values for RhB found in the literature have been determined with the same typical pulse duration of about 150 fs [19].

**Methyl 4-(6-chloro-1,3-dioxo-1*H*-benzo[*de*]isoquinolin-2(3*H*)-yl)benzoate (1):** To a solution of 4-chloro-1,8-naphthalic anhydride (2 mmol, 0.46 g) in 1,4-dioxane (10 mL), methyl 4-aminobenzoate (6 mmol, 0.90 g) was added at room temperature. The mixture was heated under reflux for 24 h. The product was filtered, purified by crystallization from EtOH, and dried. Yield: 77%, 0.56 g; pale yellow crystals, mp > 300 °C; ^1^H NMR (CDCl_3_) δ 3.94 (s, 3H), 7.39 (d, *J* = 8.8 Hz, 2H), 7.86 (d, *J* = 7.9 Hz, 1H), 7.89 (dd, *J*_1_ = 8.5, *J*_2_ = 7.3 Hz, 1H), 8.22 (d, *J* = 8.8 Hz, 2H), 8.53 (d, *J* = 7.9 Hz, 1H), 8.67 (d, *J* = 8.5 Hz, 1H), 8.7 (d, *J* = 8.5 Hz, 1H); Anal. calcd for C_20_H_12_ClNO_4_: C, 65.67; H, 3.31; N, 3.83; found: C, 65.30; H, 3.24; N, 3.79.

**4-(6-Chloro-1,3-dioxo-1*H*-benzo[*de*]isoquinolin-2(3*H*)-yl)benzoic acid (2):** Derivative **1** (1.0 mmol, 0.36 g) was suspended in water (5 mL) and a solution of sodium hydroxide (1.3 mmol, 0.05 g) in water (5 mL) was added at room temperature. The mixture was heated at 80 °C for 18 h. The homogeneous reaction mixture was acidified with 1 M HCl solution until approximately pH 2. The product was filtered, crystallized from MeOH and dried. Yield: 63%, 0.22 g; beige powder, mp > 300 °C; ^1^H NMR (DMSO-*d*_6_): δ 7.78 (dd, *J*_1_ = 8.49, *J*_2_ = 7.54 Hz, 1H), 7.81 (d, *J* = 7.74 Hz, 2H), 7.91 (d, *J* = 7.82 Hz, 2H), 8.04 (dd, *J*_1_ = 7.15, *J*_2_ = 1.30 Hz, 2H), 8.43 (ddd, *J*_1_ = 8.49, *J*_2_ = 1.22, *J*_3_ = 0.63 Hz, 2H), 13.04 (br s, COOH); Anal. calcd for C_19_H_10_ClNO_4_: C, 64.88; H, 2.87; N, 3.98; found: C, 64.54; H, 2.80; N, 3.86.

**4-(6-Hydroxy-1,3-dioxo-1*H*-benzo[*de*]isoquinolin-2(3*H*)-yl)benzoic acid (3):** Derivative **1** (1.0 mmol, 0.36 g) was suspended in water (5 mL) and a solution of sodium hydroxide (4.0 mmol, 0.16 g) in water (5 mL) was added at room temperature. The mixture was heated under reflux for 24 h. The homogeneous reaction mixture was acidified with 1 M HCl/H_2_O solution until approximately pH 2. The product was filtered, crystallized from EtOH and dried. Yield: 48%, 0.20 g; pale yellow powder, mp > 300 °C; ^1^H NMR (DMSO-*d*_6_) δ 7.54 (d, *J* = 8.69 Hz, 2H), 8.04 (dd, *J*_1_ = 8.45, *J*_2_ = 7.35 Hz, 1H), 8.08 (d, *J* = 8.69 Hz, 2H), 8.10 (d, *J* = 7.90 Hz, 1H), 8.47 (d, *J* = 7.90 Hz, 1H), 8.61 (dd, *J*_1_ = 7.27, *J*_2_ = 1.07 Hz, 1H), 8.68 (dd, *J*_1_ = 8.49, *J*_2_ = 1.07 Hz, 1H), 11.02 (s, OH); 13.04 (br s, COOH); Anal. calcd for C_19_H_11_NO_5_: C, 68.47; H, 3.33; N, 4.20; found: C, 68.31; H, 3.32; N, 4.18.

**4-(1,3-Dioxo-6-(piperidin-1-yl)-1*H*-benzo[*de*]isoquinolin-2(3*H*)-yl)benzoic acid (4): Method A**. Derivative **2** (1.0 mmol, 0.35 g) was dissolved in 1,4-dioxane (5 mL), and piperidine (2 mmol, 0.2 mL) was added at room temperature. The mixture was heated under reflux for 8 h. The basic reaction mixture was neutralized with 1 M HCl solution until approximately pH 6–7. The product was filtered, crystallized from MeOH and dried. Yield: 10%, 0.04 g. **Method B**. Derivative **6** (1.0 mmol, 0.36 g) was suspended in water (5 mL) and a solution of sodium hydroxide (1.3 mmol, 0.05 g) in water (5 mL) was added at room temperature. The mixture was heated at 80 °C for 18 h. The homogeneous reaction mixture was acidified with 1 M HCl solution until approximately pH 2. The product was filtered, crystallized from MeOH and dried. Yield: 69%, 0.17 g; orange crystals, mp > 300 °C; ^1^H NMR (DMSO-*d*_6_) δ 1.60–1.80 (m, 6H), 2.9–3.0 (m 4H), 7.36 (d, *J* = 8.1 Hz, 1H), 7.49 (d, *J* = 8.5 Hz, 2H), 7.84 (dd, *J*_1_ = 8.4, *J*_2_ = 7.3 Hz, 1H), 8.07 (d, *J* = 8.5 Hz, 2H), 8.40 (d, *J* = 8.1 Hz, 1H), 8.47 (d, *J* = 8.4 Hz, 1H), 8.48 (d, *J* = 8.1, 1H), 13.00 (br s, COOH); Anal. calcd for C_24_H_20_N_2_O_4_: C, 71.99; H, 5.03; N, 7.00; found: C, 71.41; H, 5.10; N, 6.89.

**6-(Piperidin-1-yl)-1*H*,3*H*-benzo[*de*]isochromene-1,3-dione (5):** Preparation according to [1]. The product was isolated in 82% yield, mp 174–176 °C (lit. [1] 175–176 °C).

**Methyl 4-(1,3-dioxo-6-(piperidin-1-yl)-1*H*-benz[*de*]isoquinolin-2(3*H*)-yl)benzoate (6):** 4-Piperidinyl-1,8-naphthalic anhydride **5** (1 mmol, 0.28 g) was dissolved in 2-ethoxyethanol (7 mL) and methyl 4-aminobenzoate (3 mmol, 0.45 g) was added at room temperature. The mixture was heated under reflux for 24 h. The product was filtered, crystallized from EtOH and dried. Yield: 89%, 0.34 g; yellow crystals, mp 280–282 °C; ^1^H NMR (CDCl_3_) δ 1.68–1.81 (m, 2H), 1.86–2.0 (m, 4H), 3.24–3.33 (m, 4H), 3.95 (s, 3H), 7.25 (d, *J* = 7.2 Hz, 1H), 7.39 (d, *J* = 8.2 Hz, 2H), 7.72 (dd, *J*_1_ = 8.3, *J*_2_ = 7.4 Hz, 1H), 8.21 (d, *J* = 8.2 Hz, 2H), 8.49 (d, *J* = 8.5 Hz, 1H), 8.53 (d, *J* = 8.5 Hz, 1H), 8.61 (d, *J* = 7.2 Hz, 1H); Anal. calcd for C_25_H_22_N_2_O_4_: C, 72.46; H, 5.35; N, 6.76; found: C, 72.03; H, 5.29; N, 6.80.
